# Realistic retinal modeling unravels the differential role of excitation and inhibition to starburst amacrine cells in direction selectivity

**DOI:** 10.1371/journal.pcbi.1009754

**Published:** 2021-12-30

**Authors:** Elishai Ezra-Tsur, Oren Amsalem, Lea Ankri, Pritish Patil, Idan Segev, Michal Rivlin-Etzion

**Affiliations:** 1 Department of Brain Sciences, Weizmann Institute of Science, Rehovot, Israel; 2 Department of Mathematics and Computer Science, The Open University of Israel, Ra’anana, Israel; 3 Department of Neurobiology, Hebrew University of Jerusalem, Jerusalem, Israel; 4 Edmond and Lily Safra Center for Brain Sciences, Hebrew University of Jerusalem, Jerusalem, Israel; Stiftung caesar, GERMANY

## Abstract

Retinal direction-selectivity originates in starburst amacrine cells (SACs), which display a centrifugal preference, responding with greater depolarization to a stimulus expanding from soma to dendrites than to a collapsing stimulus. Various mechanisms were hypothesized to underlie SAC centrifugal preference, but dissociating them is experimentally challenging and the mechanisms remain debatable. To address this issue, we developed the Retinal Stimulation Modeling Environment (RSME), a multifaceted data-driven retinal model that encompasses detailed neuronal morphology and biophysical properties, retina-tailored connectivity scheme and visual input. Using a genetic algorithm, we demonstrated that spatiotemporally diverse excitatory inputs–sustained in the proximal and transient in the distal processes–are sufficient to generate experimentally validated centrifugal preference in a single SAC. Reversing these input kinetics did not produce any centrifugal-preferring SAC. We then explored the contribution of SAC-SAC inhibitory connections in establishing the centrifugal preference. SAC inhibitory network enhanced the centrifugal preference, but failed to generate it in its absence. Embedding a direction selective ganglion cell (DSGC) in a SAC network showed that the known SAC-DSGC asymmetric connectivity by itself produces direction selectivity. Still, this selectivity is sharpened in a centrifugal-preferring SAC network. Finally, we use RSME to demonstrate the contribution of SAC-SAC inhibitory connections in mediating direction selectivity and recapitulate recent experimental findings. Thus, using RSME, we obtained a mechanistic understanding of SACs’ centrifugal preference and its contribution to direction selectivity.

## Author summary

Retinal direction selectivity is a canonical example for a computation undertaken by the retina. Starburst amacrine cells (SACs), interneurons in the retina, mediate direction selectivity via two mechanisms: they form asymmetric inhibitory connections with direction selective ganglion cells (DSGCs); and their processes are themselves direction selective, displaying a centrifugal preference. Various hypotheses were raised to account for this centrifugal preference, including the arrangement of SAC excitatory inputs, their kinetics, as well as reciprocal inhibition between SACs. To address this, we developed the Retinal Stimulation Modeling Environment (RSME)–a modeling environment for highly detailed, biologically plausible simulations, tailored to the exploration of neuronal dynamic and visual processing in retinal circuits. We started with exploring the excitation to a single SAC, and found that a precise organization of the input kinetics along SAC processes can generate a centrifugal preference that matched our experimental recordings. We then generated a network of SACs and found that reciprocal inhibition between SACs further enhances the centrifugal preference. Finally, we embedded a DSGC in the network, and dissected the contribution of SAC-DSGC asymmetric connections and SAC centrifugal preference to direction selectivity in DSGC.

## Introduction

**Fig 1:**
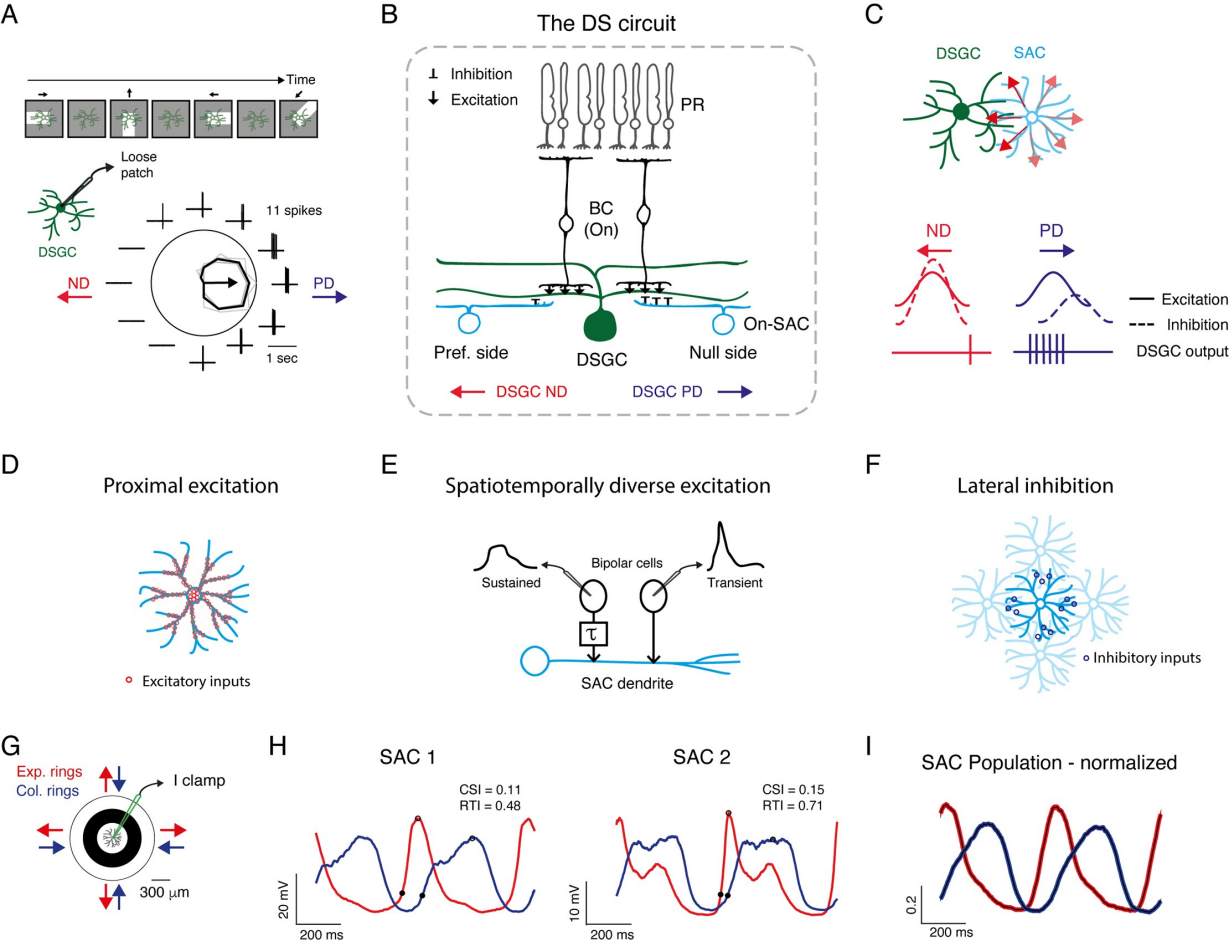
Mechanisms for direction selectivity and centrifugal preference in experimentally recorded SAC. **A.** Example directional tuning of a DSGC. Top: Illustration of a moving bar stimulation. Bottom: Spiking activity recorded in loose-patch mode. The polar plot represents the mean spike count (black) in response to the leading edge of a white bar (On response) moving in 12 directions; grey lines represent single repetitions (4 in total); the arrow represents the preferred direction. The surrounding traces depict one representative recording trace. **B.** A cross-section of the direction selective circuit. Only inputs to DSGCs from the On layer are illustrated for simplicity. DSGCs receive excitatory inputs (↓) from bipolar cells and inhibitory inputs (⊥) from SACs. SACs on the null side form stronger inhibitory connections than SACs on the preferred side. **C.** Schematic of a DSGC innervated by a null-side SAC, top view. SAC processes respond with greater depolarization to centrifugal motion (red arrows), corresponding to the DSGC’s null direction. Bottom: illustration of the excitatory and inhibitory inputs to DSGC during preferred and null motion, and their resulting spiking activity. **D-F.** Illustration of the mechanisms that are thought to underlie SAC CF preference: proximal distribution of excitatory inputs (**D**), differential input kinetics (**E**), and SAC-SAC inhibitory connections (**F**). **G.** Experimental design: current-clamp recordings were performed from On-SACs in response to expanding and collapsing rings centered on the cell soma. **H.** Two examples of SAC voltage responses to expanding (red) and collapsing (blue) rings stimulation, averaged over cycles and repetitions. Responses to two cycles of the rings are shown. Black dots denote the initial response and the peak. **I.** Normalized waveforms (mean±SEM) of all experimentally recorded SAC responses to expanding and collapsing rings averaged over 1 second (n = 27 cells). DSGC: direction selective retinal ganglion cell; SAC: starburst amacrine cell; BC: bipolar cell; PR: photoreceptor; PD: preferred direction; ND: null direction.

Retinal direction selectivity emerges in direction selective retinal ganglion cells (DSGCs), which strongly respond to motion in one (preferred) direction and weakly to motion in the opposite (null) direction (**Fig 1A**) [1–4]. The key mechanism for generating direction selectivity in DSGCs is asymmetric GABAergic inhibition from starburst amacrine cells (SACs) [5–7]. This asymmetry is achieved by asymmetric wiring from SACs to DSGCs [8] combined with the centrifugal (CF) preference of SAC processes (SAC dendrites and axons are synonymous and called processes): SAC processes respond more strongly to motion away from the cell soma (CF) than towards cell soma (centripetal, CP) (**Fig 1B and 1C**) [9–11].

Different hypotheses have been raised to account for SACs’ CF preference, including their intrinsic properties, excitatory input distribution, and their reciprocal inhibitory connections [2–4,12–14]. The intrinsic properties of the SAC, such as the differential expression of chloride transporters, voltage-gated ion channels, and somatic activation of mGluR receptors were all suggested to contribute to SAC CF preference [9,15–21].

The input distribution hypothesis is supported by two recent studies showing that SAC excitatory inputs are concentrated in its proximal 2/3 of dendritic arbors and skewed away from the distal release sites, an organization that is thought to underlie SAC CF preference (**Fig 1D**) [10,11]. In addition, physiological and anatomical data have detected different kinetics of the excitatory inputs from bipolar cells to SACs, with more sustained excitation in proximal processes and more transient excitation towards distal processes [22–24]. The precise spatiotemporal distribution may contribute to SAC CF preference, as only during centrifugal motion the sequential activation of the sustained and transient inputs is effectively integrated (**Fig 1E**). Yet, this spatiotemporal dependence of excitation has not always been observed in experimental data and its contribution to SAC CF preference remains controversial [11,25].

The reciprocal connections between SACs form a dense inhibitory network, which has also been suggested to contribute to SAC CF preference (**Fig 1F**) [26–28]. However, the role of inhibitory connections between SACs is also unsettled as blocking GABA receptors fails to fully eliminate SACs’ CF responses [15,16,29,30]. A study that combined morphologically constrained SAC network model and dendritic imaging suggested that reciprocal SAC inhibition supports velocity and contrast tuning [11]. Another recent study suggested that SAC-SAC inhibitory connections mildly affect SAC activity and are more dominant for the computation of direction selectivity in DSGCs under certain stimulus conditions, particularly when the moving stimulus is presented on a noisy background [31].

Surprisingly, despite the ample studies dedicated to identifying the source of SAC CF preference, its role in mediating direction selectivity in DSGC is not yet solved. Whereas selective reduction of SAC CF preference was found to decrease direction selectivity [29], another study rendered inhibitory inputs to DSGC symmetric (indicating loss of SAC CF preference) and direction selectivity was still maintained [30].

To find the mechanistic balances for SAC CF preference, we developed the Retinal Stimulation Modeling Environment (RSME) framework: a modeling environment for highly detailed, biologically plausible simulations tailored to the exploration of visual processing in retinal circuits. RSME is a versatile framework that supports single cell as well as network modeling. It incorporates detailed morphological and biophysical constraints of each neuron alongside providing efficient modules for retinal mosaic organization, connectivity schemes, synaptic dynamics and graded synaptic release. RSME has a module for generating structured visual stimuli, allowing to assess the responses of the simulated neurons to various light patterns. Using RSME, we aimed to reveal the network mechanisms that can generate a CF preference in simulated SACs that resembles our electrophysiological recordings. We further pushed the neuronal circuits to extreme conditions that are experimentally unfeasible to unfold the role of excitatory input kinetics arrangement and the contribution of SAC-SAC inhibitory connections to SAC CF preference and direction selectivity in the DSGC.

## Results

### Electrophysiological recordings reveal SAC CF preference in response to moving rings

We previously demonstrated SAC CF preference using patch-clamp recordings from On-SACs in the isolated mouse retina [32]. The electrophysiological SAC recordings presented here combine published and new data. The retina was presented with expanding and collapsing rings centered on the SAC soma, and SAC voltage was recorded in current-clamp mode (**Fig 1G**). In accordance with previous findings [9–11,32], expanding rings, which generate centrifugal motion, evoked a stronger and faster depolarization response in SACs than collapsing rings, which generate centripetal motion (**Fig 1H**). SAC response amplitude and kinetics dictate the amount and timing of the inhibitory currents in a postsynaptic DSGC and thereby its directional preference [32]. We therefore used two parameters to assess SAC CF preference, the Centrifugal Selective Index and the Rise Time Index (CSI and RTI, respectively, see *Methods*). The CSI is evaluated based on SAC response amplitude and positive values indicate larger response amplitudes to centrifugal motion. The RTI is evaluated based on SAC response rise time, measured as the time from the initial response (when the voltage reached 20% of the peak) to peak response [32]. Positive RTI values indicate shorter rise times during centrifugal motion than during centripetal motion. While we detected some variability between the experimentally recorded SACs (**Fig 1H**), both CSI and RTI values tended to be positive (CSI: 0.18±0.17, RTI: 0.33±0.22, mean ± STD), as is also reflected in the average voltage response to expanding and collapsing rings of all SACs (**Fig 1I**; n = 27 cells).

### Modeling environment

**Fig 2:**
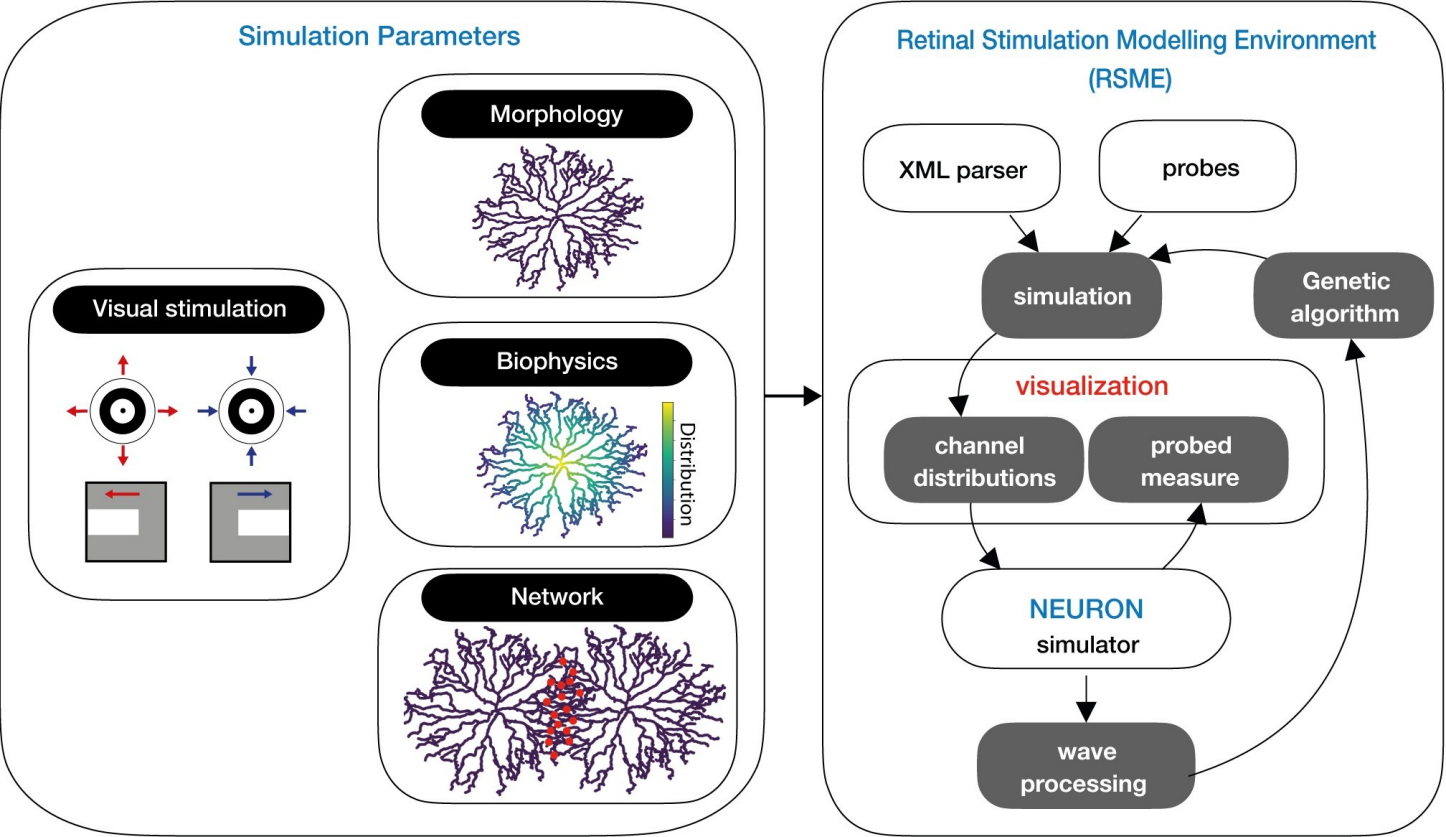
RSME schematic. RSME is comprised of model specifications and a simulation environment. Parameters include the visual stimulation pattern, cellular morphology and biophysics, and network organization. Simulation parameters are parsed and visualized within the modeling environment and used to simulate over NEURON. RSME permits the utilization of signal processing for local optimization (e.g., via a genetic algorithm).

RSME encapsulates NEURON to provide a retina-focused modeling framework in which simulated neurons can be stimulated with visual patterns. RSME comprises a NeuroML-inspired XML-based specification interface [33] and a dedicated parsing engine, which supports detailed biophysical, morphological, network architecture, and stimulation parameters. It features a set of mechanisms for retinal circuitry related specifications, including (1) graded synaptic transmission-based communication, which is essential as most retinal neurons do not produce action potentials and use graded neurotransmitter release instead; (2) orientation-based rules for synaptic connectivity to accommodate previously reported retinal connectivity patterns [8]; (3) arrangement of cells of the same type in a grid, supporting retinal mosaic organization [34,35]. Data specification (e.g., number of neurons, synaptic distribution and location) can be visualized with a dedicated module and is processed to generate a NEURON model. RSME also supports iterative execution with varying initial conditions, which can be used for model optimization, as demonstrated below using a genetic algorithm. Results are logged, saved and visualized, and are available for further analysis. A schematic of the software architecture is given in **Fig 2**. Detailed information on RSME architecture can be found in *Methods* (see **S1–S3 Figs**) and in the project’s GitHub. RSME is an open-source framework available at: https://github.com/NBELab/RSME and its detailed documentation is available at: https://elishai.gitbook.io/retinal-stimulation-modeling-environment/ [36].

### Simulating a single SAC: SAC’s spatiotemporal excitatory inputs can generate CF preference

We first utilized RSME and a genetic algorithm to test whether a set of synaptic properties can give rise to the CF preference in a single SAC [9,22,32]. For this, we used the morphology of a reconstructed On-SAC [32] (Neuromorpho.org; ID: NMO_139062), transformed it into a 3D multicompartmental model and specified the passive properties of the cell (**S4 Fig**). We used a genetic algorithm to scan for a combination of spatial organization and temporal dynamics of excitatory inputs that induce SAC CF preference (for an explanation on GA, please see **S5A–S5F Fig** and *Methods*). For each set of synaptic inputs, the simulated SAC was presented with expanding and collapsing rings. CF preference was assessed based on response amplitude (measured as CSI) and rise time (RTI), similar to the experimental data.

**Fig 3:**
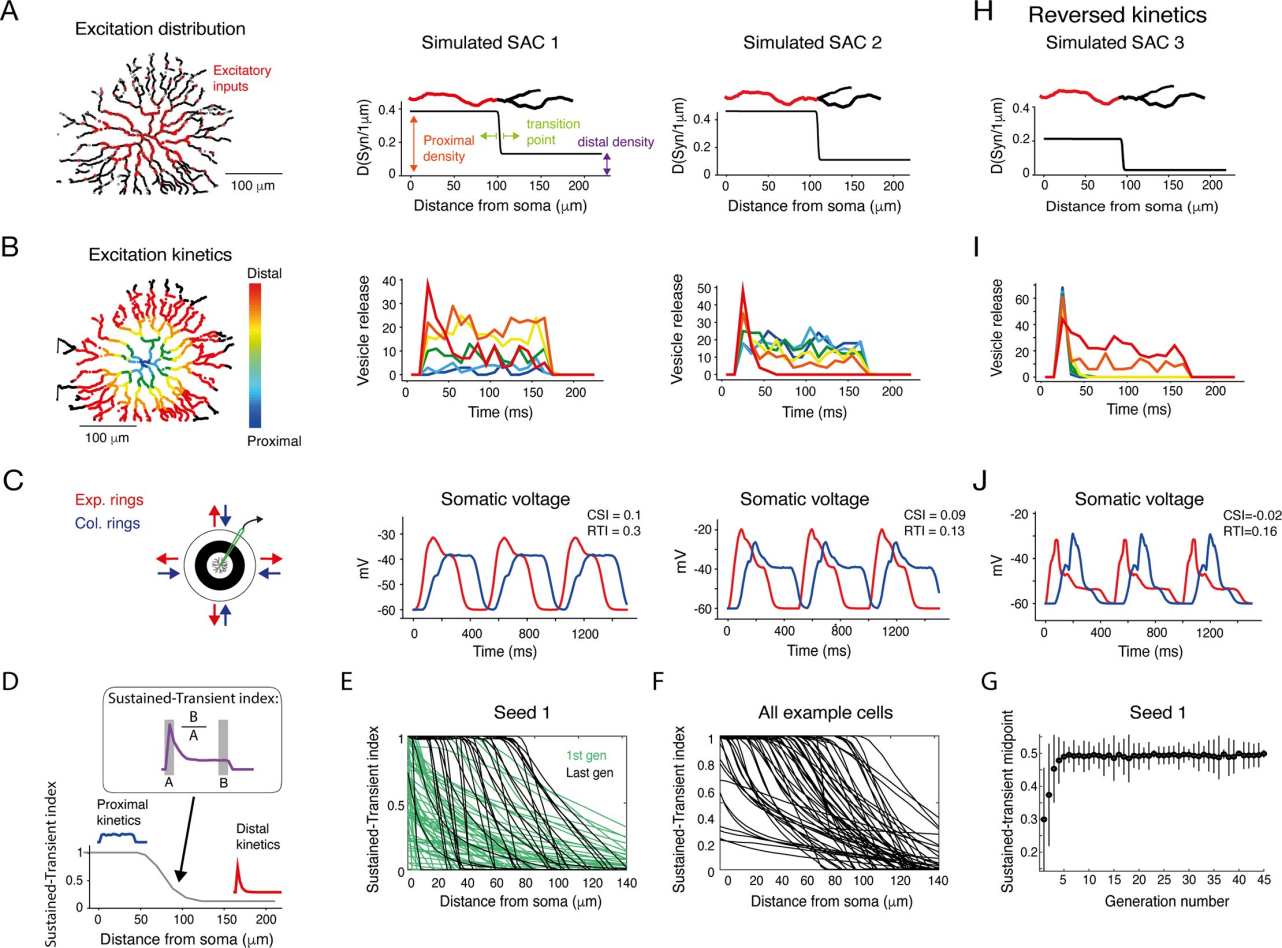
Kinetic properties of excitatory inputs can generate CF preference in simulated SACs. **A.** Left: Reconstruction of a SAC showing the spatially restricted simulated excitatory inputs (red dots). Right: Density of excitatory synapses as a function of distance from soma for two example simulated SACs. The distribution is set by three parameters: proximal synapse density, distal synapse density and the anatomical transition point. For illustration, the transition of color in the example process on top depicts the location of the anatomical transition point. **B.** Left: Illustration of the spatiotemporally diverse excitation distribution, color-coded according to distance from the SAC soma. Right: The kinetics of excitatory inputs in different locations along SAC processes are color-coded by their distance from the cell soma for the two example simulated SACs. **C.** Left: Illustration of the stimulus. Right: The somatic voltage of the two simulated SACs in response to expanding (red) and collapsing (blue) rings. **D.** The sustained-transient index of the excitatory inputs was calculated at each dendritic location based on the input kinetic waveform (see inset and *Methods*). Values of 1 and 0 indicate completely sustained and transient input kinetics, respectively. **E.** The sustained-transient index as a function of distance from cell soma for all cells in the first generation (light green) and last generation (#45; dark green) of an example simulation seed. **F.** Same as **E**, for all example cells shown in **Fig 5C and 5E**. **G.** The midpoint of the sustained-transient index for all cells in the example seed as a function of generation. By the 6^th^ generation, the indices tend to span the entire range–from 1 in the proximal to 0 in the distal processes–and the index converges on a value of 0.5. **H-J.** As in **A-C** but for an example SAC with reversed kinetics of the excitatory inputs, changing from transient inputs in the proximal to sustained inputs in the distal processes.

Our genetic algorithm explored an 8-dimensional parameter space that controls the distribution and response kinetics of the synapses. Three parameters control the distribution of bipolar cell synapses along the SAC processes according to a sigmoid function, going from denser in the proximal processes to sparser in the distal processes as previously shown [10,11] (**Fig 3A**). The distribution function was parameterized by a proximal density value (synapse probability per 1 μm), a density transition point (the distance from the soma in which input density changed; this point divides the densely innervated proximal processes from the sparsely innervated distal processes), and a distal density value (**Fig 3A**; see *Methods*). Four parameters control the kinetics of bipolar cell synapses, designed using a stochastic vesicle release mechanism [37]. These synapses were regulated by vesicle refilling rate, release probability, a kinetic transition start-point that dictates the location where the synaptic inputs start to change from sustained to transient, and a kinetic transition endpoint that dictates the area where the kinetics remain constant (See *Methods*). Based on the spatiotemporal input hypothesis, the release kinetics were set to shift gradually from more sustained in proximal to more transient in distal synapses (**Fig 3B)** [22–24]. The eighth parameter controls the conductance of the bipolar cell synapses.

Many different parameter configurations were found to produce a CF preference in the SAC, implying that various combinations of anatomical constraints on the excitatory inputs and their kinetics can lead to SAC CF preference. We used six different seeds for the genetic algorithm and found that the results were qualitatively the same, concentrated in a region of parameter space which is well-performing, independent of the initial parameters. Indeed, it was previously suggested that multiple parameter sets may often provide equally valid solutions [38,39]. The set of cells that displayed a clear CF preference, as indicated by Amp_CF_-Amp_CP_≥4, RT_CF_-RT_CP_≥0, and Voltage_score_≥0 (see *Methods*), is localized to only a subset of the initial distribution, indicating that only a smaller space of parameters produces realistic responses (**S6 Fig**). Here, the anatomical transition point provided an insight into the anatomical distribution of excitatory inputs that can generate SAC CF preference: as mentioned above, the distribution of inputs from bipolar cell synapses is confined to SAC’s proximal 2/3 dendritic arbors [10,11,32]. The genetic algorithm demonstrated that CF preference in SACs could also arise when the transition point is significantly closer to the SAC soma than observed biologically (**S6 Fig**, see *Discussion*).

Two example sets of SAC parameters are shown together with the voltage trace produced by RSME (**Fig 3A–3C**). In these examples, the distribution of inputs from bipolar cell synapses matched the known anatomical constraints of SAC excitatory inputs. These two examples resemble responses of other SACs with similar anatomical constraints and demonstrate the similarity of the results of the genetic algorithm and our experimental recordings (compare **Fig 3C** to **Fig 1H**).

We next investigated the contribution of non-homogenous input kinetics in generating SAC CF preference. First, we assessed the input kinetics along the SAC process based on the sustained-transient index of the synaptic input as a function of its location (**Fig 3D**). According to the constraints we set, the sustained-transient index decreased with distance from cell soma. However, the dynamics of this decrease differ between the first and the last generations of the genetic algorithm. In the first generation, before the simulation converged to CF-preferring SACs, the decrease in the sustained-transient index was moderate and started already at the very proximal processes. As the algorithm converged and the generations primarily comprised CF-preferring SACs, the decline of the sustained-transient index was steeper and located further from cell soma (**Fig 3E–3G**). The steeper decline probably allows for an optimal input integration during CF, but not CP, motion, and the location of the kinetic change probably allows for sufficient amounts of both sustained and transient inputs to integrate onto the SAC.

Second, we manipulated the input kinetics to follow the reversed logic by running the genetic algorithm once more. Here, transient inputs were confined to the proximal dendrites and became more sustained towards the distal dendrites. All other parameters space remained unchanged. Interestingly, under these reversed-kinetics conditions, the algorithm did not find any CF preferring SAC (**S5G–S5I Fig**). Instead, some SACs revealed a slight centripetal preference (**Fig 3H–3J**). Finally, when we run the simulation with fixed input kinetics the algorithm resulted with only four SACs (out of 2125) that were considered CF preferring according to our criteria, and all four of them barely crossed the threshold for inclusion. Taken together, our simulation results suggest that the distribution of excitatory input kinetics along SAC processes plays an important role in determining its CF preference.

### Somatic and dendritic voltage in response to circular rings resemble

**Fig 4:**
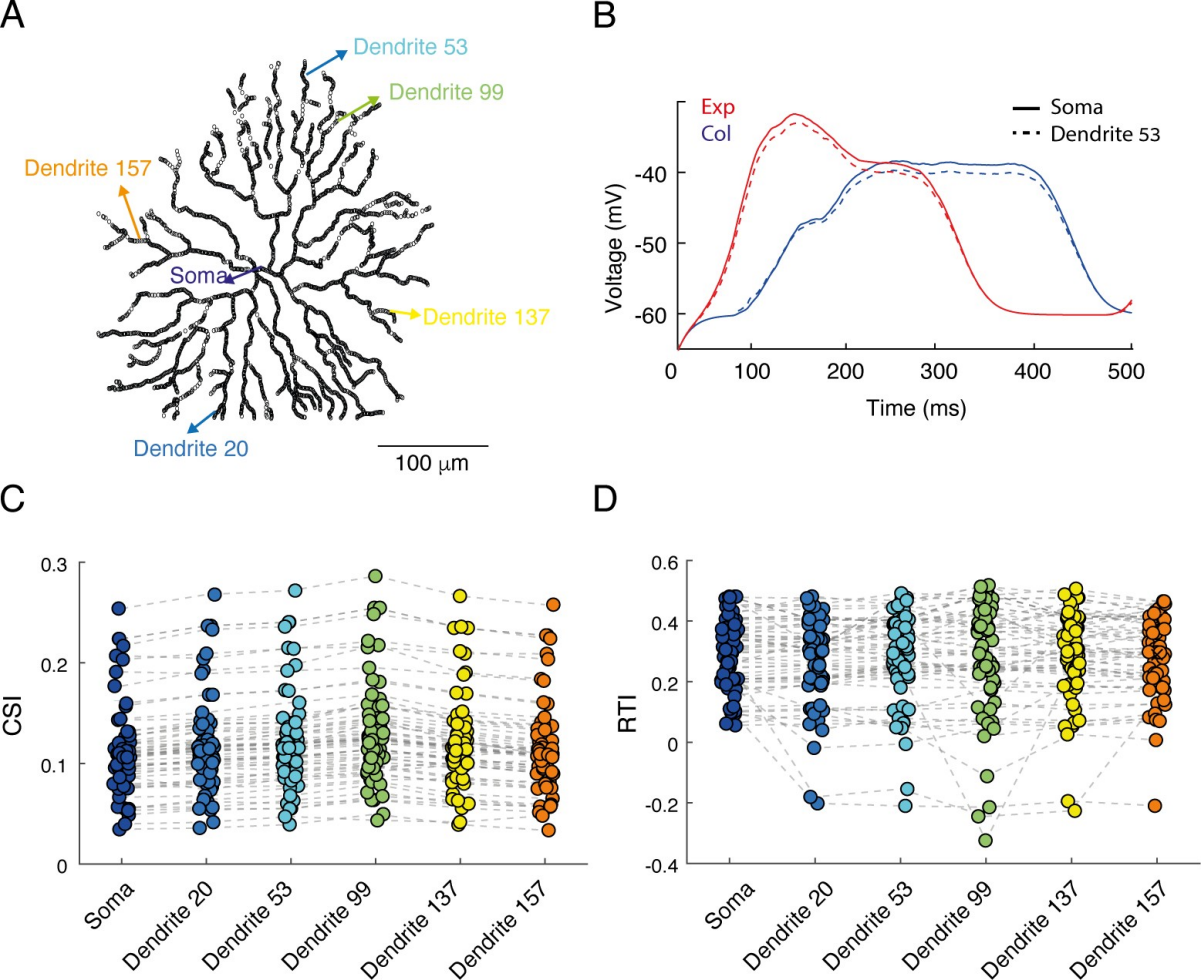
Comparison of SAC direction selectivity in the soma and distal dendrites. **A.** An image of the reconstructed SAC showing the locations used for the simulated voltage recordings, from the soma and from 5 dendritic locations. **B**. Somatic recording and the recording from one dendritic location (#53) are superimposed. **C-D**. comparison of the CSI (**C**) and RTI (**D**) values across locations. Dashed grey lines denotes values from the same cells (n = 68).

Thus far we reported on voltage responses recorded at the soma of simulated SACs. Yet, SAC’s release sites are located in its distal dendrites, and the dendritic voltage is a crucial factor that dictates the inhibitory input to the DSGC, and thereby its directional preference. The expanding and collapsing rings stimuli are suited to maximally and minimally activate all SAC dendrites, respectively, and therefore the somatic voltage is expected to reflect the correlated dendritic activity. To verify this, we ran RSME while measuring voltages at both the soma and the distal dendrites (as before, SACs included passive membrane properties but no active properties). We found that the dendritic voltage, as well as its CF preference, closely match the somatic one (**Fig 4**). We did notice a few milliseconds delay between the traces (**Fig 4B**) that is expected from the distance between the recording sites and the sequential activation of the circular rings in proximal and distal dendrites. These results are in line with a previous study that conducted simultaneous electrical recordings in the SAC soma and calcium imaging in its dendrites and showed that they are correlated [15].

### Simulating SAC network: SAC-SAC inhibitory connections enhance SAC CF preference

**Fig 5:**
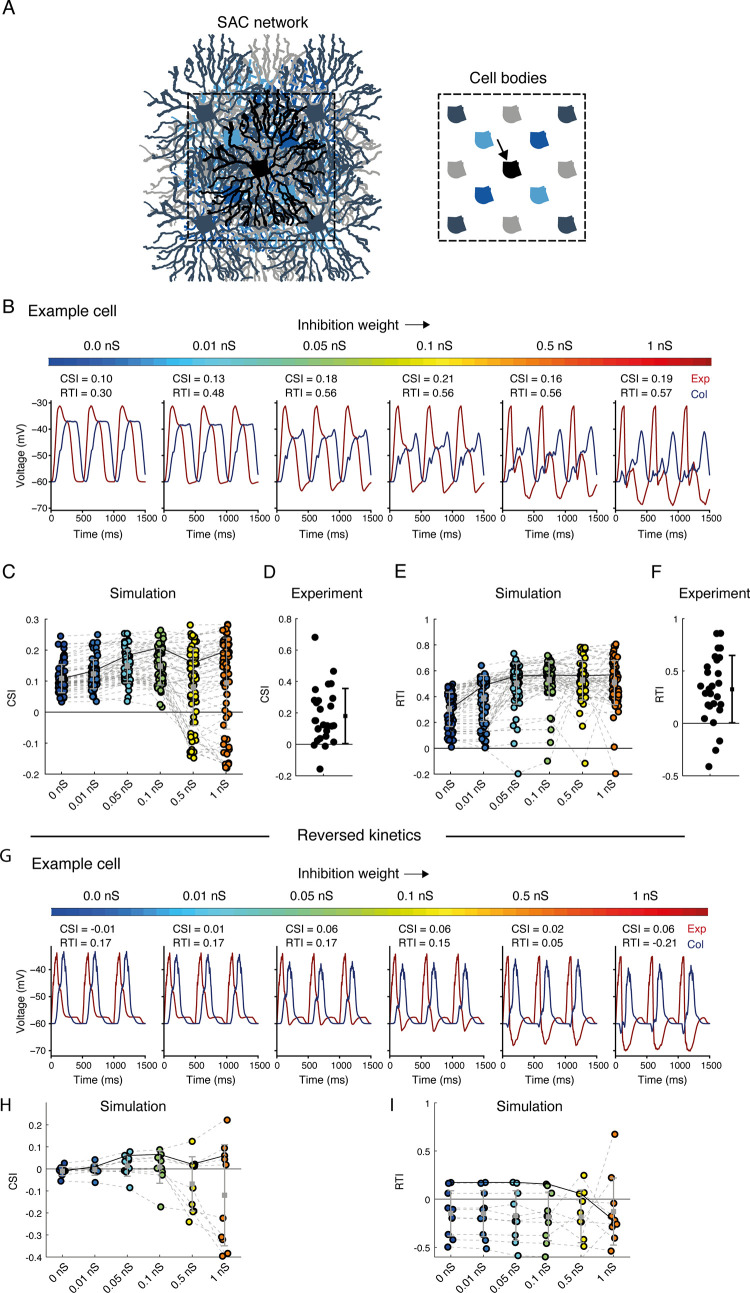
Effects of reciprocal inhibition on SAC CF preference. **A.** Simulated SAC network, consisting of two layers of replicated SAC sheets (scales of grey and blue). *Right*: illustration of all cell bodies plotted for clarification. The recorded simulated SAC (black, indicated by an arrow) resides in the center of the network. **B.** The voltage response of an example simulated central SAC (same as simulated SAC 1 from **Fig 3**) to expanding (red) and collapsing (blue) rings, shown for six different inhibition weights (colormap on top). **C.** The CSI of a population of simulated SACs (n = 76) embedded in SAC networks with different inhibition weights. Grey solid line represents the CSI values of the example SAC shown in **B**. Black solid line represents the mean CSI value. **D.** The CSI of the experimentally recorded SAC population (n = 27 cells). The dot and whiskers on the right denote mean±STD. **E.** Same as **C** but for RTI values. **F**. Same as **D** but for RTI values. **G-I.** As in **B, C, E** but for simulated SACs with reversed spatiotemporal excitation: transient in proximal and sustained in distal processes.

To investigate the contribution of reciprocal inhibitory connections between SACs to SAC CF preference, we used RSME to create networks of SACs and stimulated them with expanding and collapsing rings, which were centered on the central cell. To form these networks, we manually chose simulated SACs from the results obtained by the genetic algorithm. We restricted our choice to SACs whose set of parameters produced a CF preference (CSI>0) and obeyed the anatomical constraint of a dense excitatory input limited to the inner 1/2-2/3 of their processes in comparison with the density in the distal 1/3 dendrite [10,11,32] (n = 76 SACs). We then replicated each simulated SAC to create a two-layered design, consisting of two overlaid 3x3 and 2x2 grids of SACs (a total of 13 cells, **Fig 5A**). Within this SAC network, inhibitory GABAergic synapses were located at intersecting sections (see *Methods*), and release sites were confined to the last third of the segment [11]. **Fig 5B** illustrates the CF preference, recorded at the cell soma of the central SAC, as a function of inhibition weight between neighboring SACs in an example SAC network. Inhibition weight was determined by the conductance of the inhibitory synapses. In the absence of inhibition, the cells are independent, and the simulation returns to the initial response of a single SAC given the set of parameters found by the genetic algorithm. As depicted in the simulation example in **Fig 5B**, we found that reciprocal inhibition between SACs slightly enhanced the SAC CF preference in terms of response amplitude, and significantly enhanced CF preference in terms of response kinetics (assessed by CSI and RTI, respectively).

We then used the 76 simulated SAC networks to investigate how the strength of reciprocal inhibition in these networks affects SAC CF preference in terms of both amplitude and kinetics. We compared the CF preference of the SACs while embedded across six network configurations: in the absence of reciprocal inhibition and when inhibition gradually strengthened to 1 nS. The CSI values were positive even in the absence of functional inhibition from the network, reflecting the criteria for cell inclusion. Interestingly, the CSI values moderately increased with inhibition strength, but with stronger inhibition (>0.1 nS) the CSI values of a portion of the cells tended to decrease, and the variability within the population increased (**Fig 5C**). The CSI values extracted from the physiological data showed a clear tendency for positive values (**Fig 5D**). Searching for the source of the variability in the simulated SACs, we found that the effect of inhibition on CSI was negatively correlated with the density of excitatory inputs in the distal processes. SACs with sparse inputs in the distal dendrites tended to maintain a positive CSI, while SACs with denser inputs in the distal dendrites tended to reduce their CSI with increasing inhibitory strength (**S7 Fig**). The response kinetics of simulated SACs were more affected by the reciprocal inhibition, showing a monotonic increase in RTI values as inhibition strength increased and reaching a plateau around 0.1 nS (**Fig 5E**). The RTI values derived from the experimental data were similar to the values received in the simulations when stronger inhibition was defined (**Fig 5F**). Timing of inhibition from SAC to DSGC was shown to play an essential role in DSGCs’ directional response [30,32]. Thus, by delaying the response of SACs to CP motion, SAC-SAC inhibitory connections can delay the inhibitory input to DSGCs during preferred motion, thereby form another mechanism supporting DSGCs’ direction selectivity.

Can reciprocal inhibition between SACs per se generate CF preference? We chose non-CF preferring SACs from the original pool of cells from any generation of the genetic algorithm run to answer this. None of these SACs (n = 12 cells) displayed CF preference when reciprocal inhibition was added to the SACs network, regardless of inhibition strength, and a portion of the cells even displayed negative CSI and RTI values (**S8 Fig**). This was also true for SACs depicted from the reversed kinetic simulation (with transient proximal and sustained distal inputs, see **Fig 5G–5I;** n = 10 cells). Thus, our simulation implies that while inhibition enhances SAC CF response, it is insufficient to generate it.

### Simulating the direction selective circuit: the role of SAC-DSGC and SAC-SAC inhibitory connections in DSGC direction selectivity

So far, we have investigated the mechanisms that underlie CF preference in SAC processes. It was recently suggested that reciprocal inhibition between SACs is more influential on the DSGC directional tuning than on the SAC response amplitude [31]. We therefore shifted our focus to DSGCs, which directional response is thought to rely both on SAC CF preference and on the asymmetric wiring from SACs to DSGCs (**Fig 1**) [1–4,8]. Our goal was to dissect the contribution of each circuit component to DSGC’s direction selectivity. For this purpose, we used a reconstructed DSGC (Neuromorpho.org; ID: NMO_05318) and embedded it in a SAC network (see *Methods* for further details). In all conditions described below, the SAC-SAC inhibitory connections were either set to 0 nS (no reciprocal inhibitory connections) or to 0.1 nS (optimal strength as assessed by CSI and RTI; **Fig 5**). The SAC-DSGC inhibitory synaptic strength was fixed on 0.5 nS based on experimental data [5] (see *Methods* for a detailed explanation). To determine the threshold for spiking, we conducted intracellular current-clamp recordings from DSGCs. Based on these recordings, we set the threshold for activation to -49 mV. The baseline voltage was set to -52 mV based on measurements from DSGCs in the presence of GABA-A blockers (**S9 Fig**).

**Fig 6:**
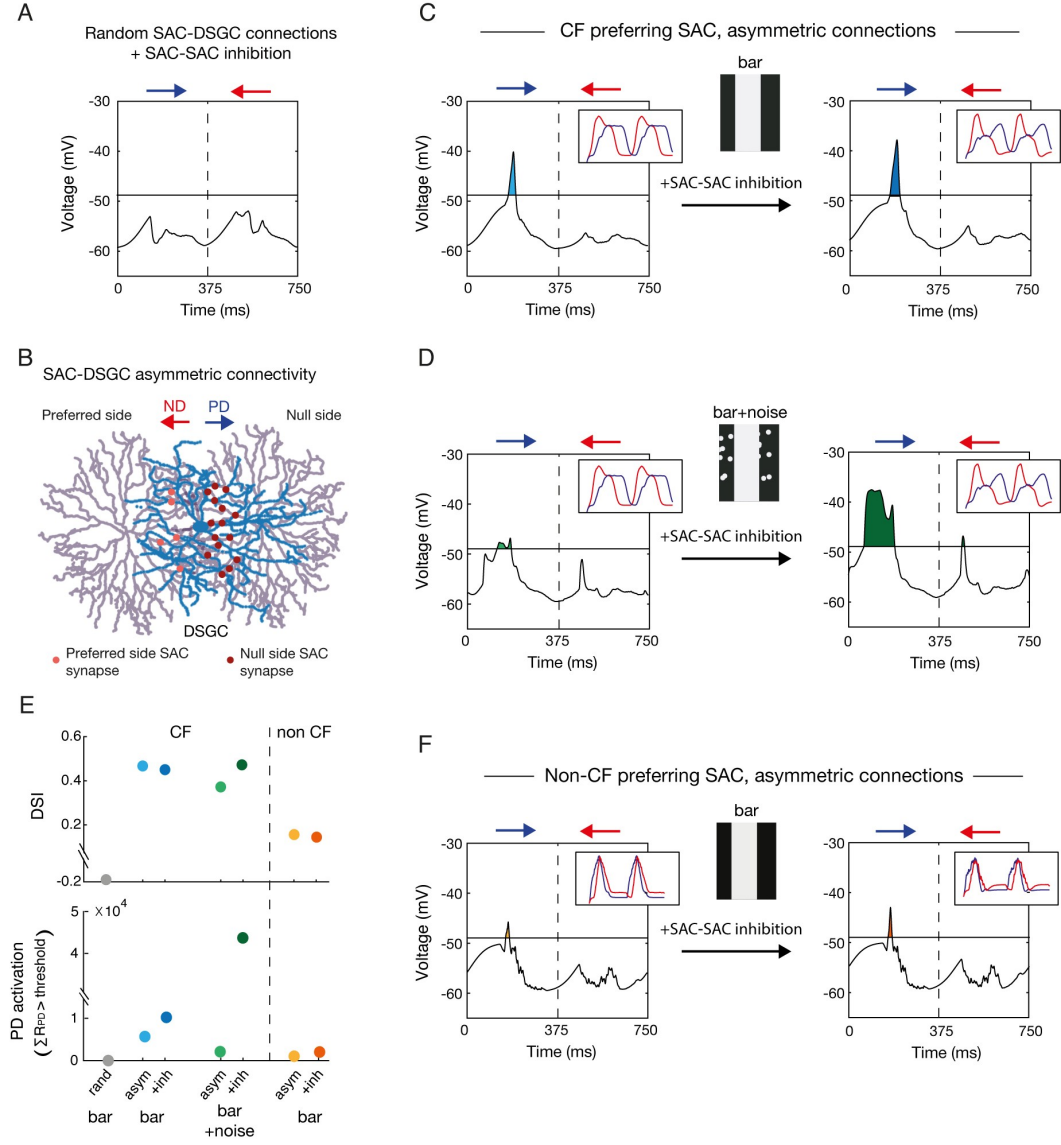
Asymmetric SAC-DSGC connections are required for DSGC’s direction selectivity. **A.** A DSGC was randomly connected to a network of CF-preferring SACs (**Fig 5A**) and consecutively presented with a bar moving in the preferred (PD, blue) and null directions (ND, red), twice in a row. Voltage traces represent the DSGC’s somatic voltage during the 2^nd^ stimulation cycle. Horizontal line represents the threshold for spiking (-49mV; see S9 Fig). **B.** Schematic of two SACs (purple) forming asymmetric GABAergic synapses (red) onto a DSGC (blue). SAC-DSGC synapses were defined with higher probability when the SAC’s process and the DSGC’s PD were antiparallel. **C.** The response of the simulated DSGC when connected to the SAC network following the asymmetric connectivity rule, in the absence (left) and presence (right) of reciprocal inhibition between SACs. Insets represent the SAC waveforms to expanding and collapsing rings in each condition. **D.** Same as **C** but when random noise was added to the background of the visual stimulus. **E.** The direction selective index (DSI) and PD activation calculated from the above simulations. The DSI is calculated according to the area under the voltage waveform (baseline to trace), PD activation is calculated as the area above spiking threshold during preferred direction motion (see *Methods*). **F.** Same as **C** but for a non-CF SAC network.

We started by a network of CF-preferring SACs that contained reciprocal inhibitory connections and were randomly connected to the DSGC. With this random connectivity scheme, the circuit did not produce direction selectivity in DSGC responses, as assessed by bars moving in two opposite directions (**Fig 6A**). Next, we implemented the known asymmetric SAC-DSGC connectivity rule, with SAC processes preferentially connecting to DSGCs with a preferred direction antiparallel to the SAC process [8]. To implement this rule, we set the probability function of synapse formation between SAC and DSGC as the inverse cosine of the similarity between the direction of the SAC process (relative to the soma) and the preferred direction of the DSGC (**Fig 6B**). When running the simulation in the absence of reciprocal inhibition between SACs, the DSGC displayed direction selectivity, as preferred direction motion evoked stronger depolarization in the DSGC than null direction motion (**Fig 6C,** left**)**. The inclusion of SAC-SAC inhibitory connections slightly increased the response in the preferred direction (referred to here as PD activation). Accordingly, the tuning strength measured by the direction selective index (DSI, see *Methods*) also increased (**Fig 6C** right and **6E** bottom).

SAC-SAC inhibitory connections were recently shown to play a unique role in direction selectivity in the presence of a noisy background [29,31]. We relied on this knowledge to further examine the biological plausibility of our model. Random flickering spots were added to the background of the moving bar (See *Methods*), and the simulation was run on the network in the absence and presence of SAC-SAC inhibition (**Fig 6D**). The addition of the noisy background did not deteriorate the directional preference, but slightly impaired the DSGC tuning. Notably, while SAC-SAC inhibitory connections somewhat improved direction selectivity in the noiseless background, their effect was stronger in the presence of noise, in line with Chen and colleagues (**Fig 6E**).

A recent study combined genetic manipulations with optogenetics to abolish SAC CF preference and demonstrated that direction selectivity in DSGCs is preserved [30]. RSME provided us with a unique opportunity to investigate the contribution of SAC CF preference to direction selectivity. For this, we generated a network of SACs from a non-CF preferring SAC (**S8A Fig**). In accordance with Hanson’s study, the direction selectivity in DSGC was overall maintained, although to a lesser degree (**Fig 6E and 6F**). This result held both in the absence and presence of reciprocal inhibition between SACs, as the latter failed to generate CF preference in non-CF preferring SACs (**Figs 6F**, inset; **S8**).

Thus, DSGC’s response was stronger in the preferred direction in all simulations except for the random connectivity condition, as reflected by the positive DSIs (**Fig 6E**). This hints that null-side connectivity of SAC-DSGC is by itself sufficient to determine the preferred direction of the DSGC. Reciprocal inhibition between SACs more strongly improved the directionality of the response when the bar was presented on top of a noisy background and had little or no effect when the bar was presented on a noiseless background or when the SAC was not CF preferring to begin with.

## Discussion

Although our understanding of the retinal connectivity pattern is rather solid [8,40,41] and an abundance of physiological data have been collected to decipher retinal function, many of the mechanisms that underlie retinal computations are yet to be deciphered. Pure experimental approaches are imperative but often insufficient to reveal the contributions of neurons’ biophysical properties, input dynamics, and network connections to their functions. Therefore, the utilization of computational tools in retina research is essential. The dynamic nature of retinal functions [42,43] and the topographic variations in visual processing [34,44] further emphasize the need for robust simulation tools to overcome uncontrolled experimental caveats. While most modeling frameworks balance the biological details with the model’s complexity, RSME was designed to provide a modeling environment for retinal circuitry, combining a high level of biological details and support for large-scale retinal circuits. RSME allows building any neuronal circuit with detailed morphology, biophysical properties, and connectivity rules. The user can generate visual patterns, stimulate the retinal circuit and acquire the voltage in each of the neurons’ compartments. RSME can be extended to model other, non-retinal neuronal circuits, and its visual stimulation module may be used to generate patterns of optogenetic activation. Here, we exploited the exploratory power of RSME to decipher the underlying mechanisms of retinal direction selectivity and the origin of SAC CF preference.

SAC CF preference is a key component in the DSGC computation and various hypotheses, from intrinsic properties to network mechanisms, have been raised over the years to explain its source. Using RSME, we were able to dissect the network mechanisms, which rely on the architecture of SAC excitatory and inhibitory inputs. In the mouse retina, excitatory inputs from bipolar cells to SAC are concentrated in the 2/3 proximal parts of its processes [10,11,32]. The genetic algorithm identified CF-preferring SACs with a matched input distribution, but it also demonstrated that CF preference could arise when the excitatory inputs are confined to an even smaller area around the cell soma. We hypothesize that biological SACs do not implement the more restricted density profile in order to maximize the excitatory receptive field area of the SAC. In addition, there is evidence that excitation to SACs is spatiotemporally diverse, with sustained bipolar cells preferentially innervating SAC proximal processes and transient bipolar cells innervating processes further from the cell soma [22,32]. EM studies identified that BC7 and BC5 contact onto Off SAC’s proximal processes and more distal processes, respectively [24]. Based on the stratification layers they suggested that the BC7 has a sustained kinetics, therefore supporting Off-SAC CF preference. Similarly, BC2 and BC3a were shown to contact onto On SAC’s proximal and more distal processes, respectively [23]. Based on physiology experiments, they concluded that this connectivity pattern also supports the CF preference in On-SACs. Note that another EM study reported similar anatomical distributions, but arrived at different conclusions, so the existence of the precise spatiotemporal input remains controversial [11], particularly for On-SACs [22,25]. Using a genetic algorithm, we demonstrated that this spatiotemporal arrangement of the excitatory inputs is sufficient to evoke SAC CF preference. When forcing the reversed arrangement on the genetic algorithm, where transient inputs innervate the proximal processes and sustained inputs innervate processes further from the cell soma, no CF preferring SAC was found. This result was obtained although we kept the anatomical constraint of denser inputs in the proximal processes. Note that both runs of the genetic algorithm could potentially find a SAC that displays fixed input kinetics, but such a solution was not found. Although we cannot exclude the option of a strong CF preference in a SAC with fixed kinetics, the fact that our algorithm run with such fixed kinetics resulted in only four CF preferring SACs (that were just above the inclusion threshold) implies a role of precise input kinetics in mediating SAC CF response. Here we used the genetic algorithm for the optimization process of CF preference in a single SAC. While the algorithm found several solutions that matched our recorded SACs, the complex parameter space does not guarantee it identified the full space of valid solutions. Other optimization approaches, such as approximate Bayesian computation and other simulation based inference methods may be implemented to overcome this limitation [45,46].

Passive models predict weak CF preference in SAC distal dendrites and a CP preference in the soma [19]. In our simulation, the precise excitatory inputs found by the genetic algorithm were enough to trigger a CF preference in the passive SAC soma, in accordance with experimental results [9,15,22,32]. In response to the circular rings, we found that the somatic and dendritic voltage closely match, in line with a previous study [15]. We expect that when using a different stimulus, which does not similarly and simultaneously recruit all SAC dendrites, the voltage recorded in different sections of the SAC will be different.

Reciprocal GABAergic inhibition between SACs have been suggested to contribute to their CF preference by reducing SAC responses to CP motion [27,47], but opposing evidence was also found [15,16,29,30]. We revealed that weak reciprocal inhibition strengthened SAC CF preference but too strong inhibition impaired the CSI values in a portion of SACs. This impairment tended to occur in simulated SACs with a relatively high density of excitatory inputs in the distal dendrites, suggesting that a sparse input density in SACs distal dendrites contributes to maintaining its CF preference in terms of amplitude in the presence of strong reciprocal inhibition. Finally, using RSME we demonstrated that although reciprocal SAC inhibition enhances SAC CF response, it cannot generate it in non-CF preferring SACs.

We tested RSME by recapitulating experimental results, showing that while inhibitory connections within the SAC network somewhat enhance direction selectivity on a uniform background, they significantly enhance direction selectivity upon a noisy background [29,31]. The rationale is that the addition of random flickering dots to the background of the moving bars effectively activates the SACs in the network such that the inhibitory connections between them are strongly engaged. Chen et al. demonstrated that the SAC-DSGC inhibitory synapse undergoes a short-term depression, which is more likely to happen on a noisy background. They suggested that the SAC-SAC inhibition prevents this depression, maintaining null-motion inhibition to DSGCs and thereby maintaining direction selectivity [31].

We also exploited RSME to test whether a network of SACs that show no CF preference can induce direction selectivity in DSGCs. Previously, selective reduction of SAC CF preference via elimination of the SACs’ GABA-A receptors was found to decrease direction selectivity in the DSGC [29]. Yet, another study that used the same elimination model with optogenetic activation of SACs showed that although inhibitory inputs to DSGCs were symmetric (indicating loss of SAC CF preference), direction selectivity in DSGCs was still maintained [30]. Our simulation results revealed direction selectivity in the DSGC even when its innervating SACs lacked any directional preference, although this selectivity was reduced compared with the network of CF preferring SACs. The positive effects of reciprocal inhibition on SAC CF preference and on the DSGC’s direction selectivity suggests a direct link between the two. Notably, our results demonstrate that asymmetric SAC-DSGC connections are essential for generating direction selectivity, as no direction selectivity emerged when SAC-DSGC connections were randomly distributed.

The extensibility of RSME enables us to further investigate the classic direction selective circuit at various mechanistic levels and thereby tackle many of the open questions in the field. First, the intrinsic properties of the SAC, such as differential distribution of ion channels along its processes [15–17,21], somatic activation of mGluR receptors [20], and the location-dependent expression of chloride transporters [18], have all been suggested to contribute to SAC CF preference. RSME uses the benefits of NEURON in the implementation of detailed biophysical properties. Each of the abovementioned intrinsic properties can be applied to the simulation, allowing the user to differentiate the role of each ion channel and transporter without compromising on the details of the whole network. Second, SAC network density was shown to be fundamental for generating direction selectivity in DSGCs [48]. Here we used a coverage factor of ~10, but SACs are known to have an even higher density in the retina and reach coverage factors of 25–70 [49,50]. RSME can control the SAC coverage factor, thereby exploring the relationship between DSGC tuning strength and SAC network density. Third, SACs release both GABA and acetylcholine. While SAC GABAergic input to the DSGC is asymmetric, the cholinergic input to DSGCs was shown to be symmetric and its role in mediating direction selectivity remains controversial [2,6,51,52]. Different studies have hypothesized that acetylcholine specializes in maintaining direction selectivity for specific stimuli, depending on contrast level, stimulus size, background, or spatial frequency [29,51,53–55]. RSME allows the implementation of acetylcholine as additional output from SACs, independent from its GABAergic output. Its visual stimulation module can generate and explore a battery of stimuli and determine the contribution of the cholinergic signal to direction selectivity in different conditions. Forth, it was shown that direction selectivity is maintained over a wide input range, such as various speeds or light intensities [12,31,56]. RSME visual stimulation module can be further used to vary the characteristics of the moving stimuli to dissect the set of mechanisms that enable a stable directional response. Fifth, while asymmetric inhibition is thought to be the key mechanism for DS, directionally-tuned excitation to DSGCs was also reported [28,51,52,57–59]. RSME can be used to implement a directional excitatory input to the DSGC and test its contribution to the DSGC’s directional preference. Sixth, while we used a relatively simplified model of the ribbon synapse to reduce the number of parameters we optimized for in the genetic algorithm, it is possible to incorporate other models in RSME, that better account for the known complexity of ribbon synapses [60,61]. Finally, the segregation to On and Off pathways is fundamental to the retinal structure. Here, we ignored the Off layer and investigated direction selectivity in the On pathway under the assumption that On and Off inputs are independent and mirror-symmetric. Yet, accumulating evidence hints towards discrepancies in the computations of the On and Off pathways [1], and there is specific evidence that reciprocal inhibition between SACs, as well as inhibition arising from wide-field amacrine cells, differentially affect On and Off direction selective computations [29]. RSME supports the implementation of another layer activated by light decrements, allowing testing the consequences of routing signals from both layers onto a single DSGC.

RSME provides a versatile, accessible framework dedicated to retinal research. Previous models of the retina used various levels of abstraction. Theoretical models have helped explain the retina’s neuronal diversity [62,63]. Linear-nonlinear (LN) models have successfully reproduced the firing rates of retinal ganglion cells [64,65]. Generalized integrate-and-fire (gIF) models have captured the influence of spike history and accounted for variability in retinal responses [66]. Realistic compartmental models have elucidated the contribution of ion-channels and morphological properties to retinal computations [11,67]. Each of these approaches advances our understanding of the system. Indeed, end-to-end dissection of retinal functions and their underlying mechanisms requires multiscale models: combining circuit connectivity and synaptic dynamics with realistic morphology and biophysics. Although various modeling frameworks have attempted to simulate such models [68–71], it has been challenging to study the biophysical mechanisms of retinal function in the context of circuit-level interactions. RSME incorporates retinal neural networks with precise synaptic connections, detailed morphological, biophysical and topological constraints of each neuron, and allows implementing various visual inputs. Together, these make RSME an attractive tool for investigating the abundance computations the retina performs, from light adaptation via direction selectivity to approach sensitivity and motion prediction [72] and evaluate their underlying mechanisms. RSME is available online at https://github.com/NBELab/RSME [36] and it is documented in a GitBook available at: https://elishai.gitbook.io/retinal-stimulation-modeling-environment/.

## Methods

### Ethics statement

All experimental procedures were approved by the Institutional Animal Care and Use Committee (IACUC) at the Weizmann Institute of Science.

### Animals

Trhr-EGFP mice (http://www.mmrrc.org/strains/30036/030036.html) [73] and mGluR2-EGFP mice [74] were used for recordings from DSGCs and SACs, respectively. Mice were from either sex and various ages, from 4 weeks to 12 months.

### Electrophysiological recordings

Dark-adapted mice were anesthetized with isoflurane and decapitated. The retina was extracted and dissected in oxygenated Ames medium (Sigma, St. Louis, MO, USA) under dim red and infrared light. The isolated retina (dorsal part) was then mounted on a 0.22 mm membrane filter (Millipore) with a pre-cut window to allow light to reach the retina and put under a two-photon microscope (Bruker, Billerica, MA, USA) equipped with a Mai-Tai laser (Spectra-physics, Santa Clara, CA, USA) as previously described [32,75]. For SAC recordings, we used mGluR2-EGFP mice [74] and for DSGC recordings, we used TRHR-EGFP mice [73], which express GFP in SACs and posterior preferring DSGCs, respectively. GFP-expressing cells were targeted for recordings with the laser wavelength set to 920 nm to minimally activate photoreceptors using a 60x water-immersion objective (Olympus, Tokyo, Japan). The isolated retina was perfused with warmed Ames solution (32–34°C) and equilibrated with carbogen (95% O2:5% CO2).

Current-clamp recordings from both SACs and DSGCs were made using 5–9-MΩ glass pipettes containing (in mM): 110 KCl, 2 NaOH, 2 MgCl2, 0.5 CaCl, 5 EGTA, 10 HEPES, 2 ATP, 0.5 GTP and 2 Ascorbate (pH = 7.2 with KOH; Osmolarity = 280). Extracellular spike recordings from DSGCs were made in loose cell-attached mode using a pipette filled with Ames solution. Data were acquired using pCLAMP10, filtered at 2 kHz and digitized at 10 kHz with a MultiClamp 700B amplifier (Molecular Devices, CA, USA) and a Digidata 1550 digitizer (Molecular Devices). The electrophysiological data of SAC recordings presented here combines published and new data [32]. All DSGC recordings are new data.

### Experimental light stimuli

Visual stimuli were generated using MATLAB and the Psychophysics Toolbox. Stimuli were projected to the retina by a monochromatic organic light-emitting display (OLED-XL, 800 x 600 pixels, 85 Hz refresh rate, eMagin, Bellevue, WA, USA) as previously described [32]. SAC CF preference was assessed by the presentation of expanding and collapsing rings centered on the SAC soma, projected via a 20x objective for 5 sec and repeated five times in a pseudo-random order. The spatial frequency of the rings was 450 μm/cycle and the temporal frequency was 2 Hz, resulting in 900 μm/sec velocity. The SAC soma was masked by a 25 μm radius grey spot [9] and the first cycle was removed from the analysis. DSGC directional tuning was assessed by its responses to bars moving in the preferred and null directions (900 μm/sec; 300 μm width; 1200 μm length) repeated four times in a pseudo-random order. DSGCs’ spike properties were extracted from their response to a 2-sec static bright spot (100 μm diameter) centered on the cell soma and projected through a 60x objective.

### Data analysis

SAC tuning was assessed by their CF preference index (CSI):

$$CSI=\frac{R(exp)-R(col)}{R(exp)+R(col)}$$

where R(exp) and R(col) are the amplitudes of the response to expanding and collapsing rings, respectively. SAC response kinetics were assessed using the response rise time, measured as the delay between the initial response (20% of the peak) and the maximum point [32]. The 20% increase was chosen rather than the 0 time point as it is probably a better estimation (although arbitrary) of the initial effective transmission. The peak timing was chosen as it represents the time of maximal inhibitory release. The rise times were then used to calculate the rise-time index (RTI) as a measure of directional asymmetries in SAC response kinetics:

$$RTI=\frac{rt(col)-rt(exp)}{rt(col)+rt(exp)}$$

where rt(col) and rt(exp) are the rise times calculated in response to collapsing and expanding rings, respectively. Similar measurements were used for experimental and simulation data.

To calculate the parameters of DSGC’s spiking, we measured the baseline voltage of the cells as the mean voltage 0.5 sec before spot illumination following the removal of fast-spiking events. Spikes evoked during the 2 seconds of light presentation were detected and removed, and the remaining voltage trace was filtered (Savitzky-Golay filter, 3^rd^ order). Traces that showed a baseline >-45 mV were removed. The spiking threshold was calculated as the maximum depolarization on which the evoked spikes were riding. 151 spikes were detected for this analysis, from 15/16 intracellularly recorded DSGCs: one cell was removed from the study due to low spiking amplitudes (<50 mV).

For simulated DSGCs, direction selectivity was assessed by two parameters: direction selective index (DSI) and PD activation. DSI was evaluated by:

$$DSI=\frac{R_{PD}-R_{ND}}{R_{PD}+R_{ND}}$$

where *R*_*PD*_ and *R*_*ND*_ are the areas between the voltage trace and -60 mV in the preferred and null direction, respectively. Note that we chose to include the subthreshold activity in this measurement as in most cases the null motion evoked no suprathreshold events and this would normally lead to a DSI of 1 and prevent appreciating the tuning sharpness in the different conditions. PD activation was measured as the area under the voltage trace and above the spiking threshold in the preferred direction. The threshold was set to 11 mV above minimum voltage to fit with our experimental measurements.

The sustained transient index (STI) was calculated for each synapse by simulating its dynamics based on release probability and refilling rate for 250 ms. Since the synapses are stochastic, we simulated each synapse 50 times and averaged the peristimulus time histogram (PSTH) (with 10 ms bins). Using the PSTH, we calculate the sustained transient index:

$$STI=\frac{steady\;state}{peak}$$

where the peak was set to the value in the first bin after the simulation starts, and the steady-state was assigned to the value in the last PSTH’s bin.

We made a 2d interpolation table for the sustained transient index over the entire range of release probabilities (1.56 × 10^−4^, 0.25) and refilling rates (5.12 × 10^−5^, 1.0) with 51 equally logarithmically spaced points along each axis. We estimated the index for each synapse by first calculating its release probability and refilling rate (both depend on distance from the soma) and then using linear interpolation over the table.

### RSME framework

RSME encapsulates NEURON [76] and its XML-based specification interface was inspired by NeuroML and used SBML (Systems Biology Markup Language) for the description of mathematical models [33,77]. RSME initialization follows a top-down approach and is defined in 5 layers:

- **Meta parameters**. Simulation unique identifier, simulation duration, and XML file paths for layers II-V. Model parametrization is based on a series of four XML files, providing input for stimulation patterns as well as architectural, morphological, and biophysical specifications (**S1 Fig**). The simulation duration used in this work was 1,500 ms.
- **Visual stimulation**. Stimulation parameters include stimulation type (e.g., moving bars or circular moving rings), stimulation field (size and location), spatial and temporal frequencies (when applicable) and duration. The stimulation pattern is parsed and conveyed into a gray-scale array, specifying the illumination level for each point in space and time (**S1 Fig**, right). The visual stimulation is transferred to the cells via the light- and dark-activated synapses (see Biophysical properties).
- **Network architecture**. Inspired by NeuroML, RSME uses *populations* of identical neurons, assigned with cell ID (defined in layer IV) and spatial arrangement. An arrangement can be specified by indicating a location in 3D space for each cell or using a template for an entire cell population. For example, cells can be arranged in a grid or layers of grids, defined by grid location, the number of cells and spacing. RSME supports multiple *populations* in a single run, and connectivity rules can be defined within a population of neurons (termed *intra-synapses*) or between *populations* of neurons (termed *projections*). Synaptic connections obey the connectivity rules and are restricted to intersections between the pre- and post-synaptic cell. Intersections are identified based on spatial proximity within a threshold value. Note that a simulation may contain all types of retinal neurons, starting with photoreceptors and ending with retinal ganglion cells. However, depending on the investigated cells and circuits, one may omit the *populations* of photoreceptors and even bipolar cells to speed up simulation run-time. In this case, synaptic inputs to the simulated cells should be designed to mimic input from bipolar cells (**S1 Fig**). The simulations described in this work used this option.
- **Cell morphology**. Each cell’s morphology is assigned with a unique identifier (ID). Morphology for each cell can be either defined using a raw reconstruction file (asc, swc filetypes) or precompiled morphology reconstruction (NEURON Hoc file). Since cell reconstruction tools often have a limited resolution, RSME supports morphology ad hoc correction functions.
- **Biophysical properties**. Each cell population is defined with its ion channels and cellular properties, including cytoplasmic resistivity, membrane capacitance and conductance dynamics of the various channels. Each channel can be distributed either uniformly or following a density function. All mathematical functions (e.g., density and distribution functions) are defined using the Systems Biology Markup Language (SBML), a widely accepted standard for bioinformatics [77]. For example, one can electronically isolate the soma with low potassium conductivity or define a high conductance of Nav1.8 sodium channels in distal dendrites (**S2B Fig**). This layer is also used to specify synapse properties. RSME supports retina-tailored specifications such as light- and dark-activated synapses. Dark-activated synapses can be defined directly on photoreceptors, mimicking the photoreceptor’s dark current. When the simulation omits photoreceptors and bipolar cells, light- and dark-activated synapses can be defined to mimic the input coming from On and Off bipolar cells, respectively. In this case, the distribution of the light- and dark-activated synapses should follow the anatomical inputs from bipolar cells, and their activation is determined by the spatiotemporal pattern of the visual stimulation. Since different bipolar cells show different response kinetics [78], RSME enables the definition of each synapse’s transient/sustained properties, indicating the temporal dynamics of the response relative to stimulus presentation.

RSME implements a flexible visualization tool for model exploration. 2-dimensional morphological projections can be visualized for each layer in either full (all sections are shown) or soma mode (where only somas and bounding boxes are shown, **S3 Fig**). Cell morphologies with synapse locations and weight distribution can also be visualized so that each cell in the model can be visualized as a whole–from cytoplasmic resistance to channel distribution (**S2 Fig**). RSME can generate a series of images listing all defined properties for each cell with a single command line. See the project’s Github for further information.

### Single-cell SAC modeling

To assess the CF preference of simulated SACs, we generated a visual stimulation pattern of expanding and collapsing rings. The stimulation field was set to 315 x 315 μm, and the rings’ position was centered on the cell soma; in our simulation, this location was x = 135 μm and y = 125 μm. Stimulus frequency was set to 2 Hz. Visual stimulation was defined to operate via bipolar cell synapses. These functions, called *alternating_expanding_circles* and *alternating_collapsing_circles*, are provided in the *Stimuli_visual_pattern* library, which we designed as part of RSME. These visual patterns are defined using functional programming: the functions calculate the phase of the current stimuli and return another function, which given a phase and location, returns the appropriate illumination value. For expanding rings, the illumination phase is defined as (*t*−*delay*)*mod*(2∙*time*_*c*_)<*time*_*c*_, where *t* is the current simulation time, *delay* is the prespecified delay time for the stimulation (set to 0 in our simulation), *time*_*c*_ is half the stimulation period, and *mod* is the modulus operator. The illumination phase is similarly defined for collapsing rings with the < operator changed to >. Within an illumination phase, we defined an effective circular field by scaling the stimulation field by $\sqrt{2}$ to account for the fact that a circle should expand far enough to cover a squared area of illumination. Other supported features are given in the project’s documentation file. Here, network specification included a single instance of a SAC, defined in the morphology specification file. We also defined an ad-hoc morphological correction function, which divided all sections’ radii by two to correct mislabeled morphology tracing.

SAC cytoplasmic specific resistance was set to 75 ohm*cm and specific capacitance was 1 μF/cm^2^. Section’s conductance was set to 0.00006 S/cm^2^ and resting potential to -60 mV. These parameters resulted in input resistance of 84 MΩ, within the experimental range observed in SAC neurons (**S4 Fig**). Each SAC had 1013 compartments.

Bipolar cell synapses onto the SAC neurons were modeled using a double exponent function (NEURON’s Exp2Syn, [76]) with a rise and decay time of 0.89 ms and 1.84 ms, respectively [79] and reversal potential of 0 mV. The synaptic conductance of each synapse was set to determine the connectivity strength. The simulation we used included On SACs and their inputs from On bipolar cells. During light offset, each synapse recruited 70 vesicles available for release [37]. During illumination, vesicles were released at each time step from this pool with some probability (*p*), and the pool size decreased accordingly. In parallel, the pool was replenished with a refilling rate (*r*). Each vesicle release results with an activation of the BP-SAC synapse. Once the visual signal changed from light to dark, the readily releasable pool of each synapse was reset to 70. For each synapse, *p* and *r* were set by the following functions:

$$p=min\left\{k\_transition\_start\_point+release\_probability*(\frac{d}{m}),1\right\}∙V_{intensity}$$

$$r\left\{ \begin{array}{cc} k\_transition\_start\_point∙refilling\_rate+refilling\_rate∙(1-\frac{d}{m}), & k\_transition\_start\_point<\frac{d}{m}\\ refilling\_rate & k\_transition\_start\_point>\frac{d}{m} \end{array}\right.$$

where *d* is the location of the synapse (0–210), *m* is the kinetic transition endpoint (*k*_*transition*_*end*_*point*), *V*_*intensity*_ is the intensity of the visual input ∈[0,1], *refilling*_*rate* and *release*_*probability* are parameters that set the starting set point for *p* and *r*, and *k*_*transition*_*start*_*point* (kinetic transition start-point) dictates how *p* and *r* change with distance from the soma. The resulted kinetics were sustained-release around cell soma, and a gradual shift from sustained to transient at a distance *k_transition_start_point* from cell soma, which continued to change up to *k_transition_end_point*, from which the most transient kinetics is kept constant. For the reversed kinetics, we set *d* to *210-d*.

The biophysical specification included bipolar cell synapse distribution and passive parameters. The distribution of the bipolar cell synapses was defined (per μm) using the following rule:

max{1−(scaling_factor*(0.5*(1+tanh(x−anatomical_transition_point)))+offset),0}

where *x* is the distance between a particular section and the soma. *scaling*_*factor*, *anatomical*_*transition*_*point* and *offset* are paramteres. The density at the soma (proximal density) is obtained by *x = 0*, and the density at the distal process is simplified to:

1−(scaling_factor+offset)

The values of these parameters were tuned using the genetic algorithm to maximize SAC CF preference in response to expanding and collapsing rings.

### Genetic optimization

We used a genetic algorithm to maximize SAC CF preference. GAs are used to provide optimized solutions to intricate nonlinear and nonconvex problems. A genetic algorithm usually comprises four genetic operators: crossover and recombination, mutation, and selection. Driven by natural selection, a group (or generation) of possible candidate solutions evolves toward better solutions (examined using some objective function). The key parameters involved are population size, the probability of crossover, the probability of mutation, the selection method and the number of generations [80].

We implemented the genetic algorithm using DEAP [81], the parameters for the first generation were selected from the following boundaries, but parameters were allowed to exceed these boundaries in the following generations if feasible according to biology (e.g., *anatomical_transition_point<dendritic length*):

refilling_rate=(0.01,1)[vesiclesperms]

release_probability=(0.01,0.75)

k_transition_start_point=(0.01,0.5)[fractionofdendriticlength]

k_transition_end_point=(2,210)[μm]

synapticconductance=(0.0001,0.2)[nS]

anatomical_transition_point=(2,210)[μm]

scaling_factor=(0.01,0.4)

offset=(0.1,0.99)

The target of the GA was chosen based on manual inspection and was set to maximize the following three objectives: (1) The difference between the maximal voltage that the SAC neuron reached during the centrifugal stimulus to that from the centripetal stimulus: Amp_CF_-Amp_CP_. (2) The difference between the rise time for the centripetal response and the centrifugal response (rise time defined as the time from 20% of the peak to the maximum point): RT_CP_-RT_CF_. (3) The difference between the maximal voltage for the centrifugal stimulus and -10 mV. If the maximal difference was below -10 mV, this objective was set to zero. This criterion was meant to prevent non-physiological depolarizations above -20 mV. Additionally, in individuals where the difference between the maximal voltage for the centrifugal and the centripetal stimuli was lower than 0.04 mV, all objective values were set to -50. The weights of objectives 1–3 were 1, 0.3, and 0.08, respectively.

We started the genetic algorithm with a population of 100 individuals and ran it for 20–45 generations. The crossover and mutation probabilities were set to 0.4. For the crossover operation, we used mutFlipBit [81]. The mutation operation was applied by replacing the mutated variable with a variable sampled from a normal distribution with a mean and standard deviation equal to the mutated variable and half of the mutated variable, respectively. We used three different selection algorithms, Non-dominated Sorting Genetic Algorithm-II [82], Indicator-Based Evolutionary Algorithm [83], and one in which the individuals were ranked according to their total score. In one case, we run the algorithm with a fixed *k_transition_end_point* = 135 μm. All three algorithms produced similar results.

### SAC network modeling

We tested the SAC network with expanding and collapsing rings. Both visual stimulation patterns were defined similarly to the single-cell simulations except for the stimulus’s center, which was set here to x = 260 μm and y = 250 μm to match the location of the central SAC in the network. The network was specified as two overlaying grids of cells. The first grid of cells was defined as a 3x3 2D grid (9 cells), with the bottom-left cell located at (0,0,0), and all other cells were organized with a 125 μm spacing between neighboring cell bodies horizontally and vertically. The second grid of cells was defined as a 2x2 2D grid (4 cells), where the bottom-left cell was cornered at (62,67,0) μm coordinate. Spacing between the cells was identical to the first grid. Overall, the network featured 13 SACs and the responses of the SAC in the center were analyzed and used in this study. Overall, we generated 76 networks, each based on a single well-performing SAC that resulted from the genetic algorithm and followed the anatomical constraints. For each network, all SACs had the same input distribution and kinetics.

SAC-SAC GABAergic connections were modeled using a double exponent (NEURON’s Exp2Syn point process). Each synapse’s rise and decay times were 3 ms and 30 ms, respectively, and the reversal potential was -75 mV. When the voltage at the presynaptic section increased above -50 mV, the synapse was activated at 200 Hz. This parameter was set to match experimental results by Lee and Zhou who measured the inhibitory current in a postsynaptic SAC while clamping a presynaptic SAC to different voltages [27]. There, clamping the presynaptic SAC to -40 mV evoked a current of ~150 pA when clamping the postsynaptic SAC at -5 mV. When we repeated these experiments on different pairs of SACs in the simulation with the above parameters and 0.1 nS conductance, we found that the required current to clamp the postsynaptic SACs was 196±130 pA (mean±std), in the range of the experimental results.

### SAC-DSGC modeling

The SAC-DSGC model was defined similarly to the SAC network specified above, with the addition of a single DSGC which was centered at (0,0,0). We only used the On layer to simplify the simulation. The DSGC’s passive parameters were identical to those of the SAC, excluding the resting potential which was set to -52 mV (**S9 Fig**). The DSGC had 1013 compartments and was defined with NEURON’s hoc file (available through Neuromorpho.org; ID: NMO_05318).

The visual stimulation in this case was a bar moving in the predefined preferred and null directions, twice in a row, with and without static noise in the background. Similar to the expanding and collapsing rings, the *alternating_bar* function returns a function that calculates the illumination level for each point in the network space at any given time. The bar’s length was 250 μm and its width was 600 μm; its motion velocity was set to 1000 μm/sec; and an x perimeter defined the area in which the bar is moving (here, 500 μm). The bar’s leading-edge location was calculated using *t*∙*velocity* and the location of its trailing edge was calculated by adding the bar’s length to the leading-edge location. The bar was set to change its direction every (x perimeter + bar size)/bar velocity. To generate a visual stimulus where spots randomly flicker in the background of a moving bar, we defined a configurable mechanism for spots generation. Specifically, we regenerated 30 spots (25 μm in radius), randomly distributed across the field of view, in each flickering phase (15 Hz). For comparison between responses to preferred and null motion, we excluded the response to the first cycle which includes the response to the appearance of the stimulus (**Fig 6**).

Note that the SACs and DSGC are not logically co-located in the same coordinate system. However, we can logically align them as we connect the two populations with synapses. Synapse formation was based on the x-y intersection (minimal distance of 5 μm) and the probability function of synapse formation between SAC and DSGC was set to the inverse cosine of the similarity between the direction of the SAC process (relative to the soma) and the preferred direction of the DSGC. The logical alignment was defined at x = 135 μm and y = 100 μm (so the DSGC’s soma was aligned to the soma of the central SAC).

The conductance of the SAC-SAC inhibitory synapse was fixed on 0.1 nS based on our simulation results (**Fig 5**) and in accordance with Lee & Zhou, who measured inhibitory conductance between pairs of neighboring SACs [27]. Here, we assumed ~20 contacts between neighboring SACs. SAC-DSGC inhibitory synapses had a reversal potential of -60 mV and the conductance was fixed on 0.5 nS based on experimental data: Wei and colleagues reported a ~9 nS inhibitory conductance between null-side SAC and DSGC pairs [5]. Assuming ~14 contacts between pairs [5], each synapse is estimated to be ~0.6 nS, which is in the range of the synaptic conductance we used. Parameters for the SAC were chosen from the results of the genetic algorithm (see simulated SAC 1 in **Figs 3** and **5B**), defined with

refilling_rate=3.7vesiclesperms

release_probability=0.08

k_transition_start_point=0

k_transition_end_point=135

synapticconductance=0.0025nS

anatomical_transition_point=102μm

scaling_factor=0.254

Offset=0.6144

### Runtime, data processing and visualizations

We used MATLAB, python and NumPy [84] for data processing. Figures and neuron morphology visualizations were created using Matlab, Matplotlib [85], seaborn [86], BlenderNEURON, and processed in Adobe Illustrator.

With a conventional MacPro featuring 2.6 GHz 6-Core Intel Core i7, 16 GB 2400 MHz DDR4 memory, and Intel UHD 630 1536 MB Graphics card, we measured a simulation runtime of: 70.659 sec for a single SAC, 421.465 sec for the SAC network, and 492.357 sec for the SAC network with the DSGC. Please note that this runtime was measured after precompiling the visual stimulation data to determine the visual input to each neuronal compartment at each time step. On first runs, this compilation of visual data must be obtained. We measured a visual stimulation compilation time of 612.862 sec for a single SAC, 1216.572 sec for a network of SAC and 1503.471 sec for the SAC network with the DSGC.

### Computer simulation

All simulations were run using NEURON 7.7 [76] with step time of 0.025ms. Genetic algorithm simulations were performed on the Blue Brain V supercomputer based on the HPE SGI 8600 platform hosted at the Swiss National Computing Center in Lugano, Switzerland. Each compute node was composed of an Intel Xeon 6140 CPU @2.3 GHz and 384 GB DRAM. All other simulations were performed on a 2.6 GHz 6-Core Intel Core i7, 16GB RAM, Mac computer.

## Supporting information

S1 Fig RSME top-down approach toward modeling. Modeling is initiated with XML-based specification. The visual stimulation pattern is precompiled to improve run time over multiple simulation runs. Parameters are parsed within RSME, which initializes a logger and supporting data entities and creates the model. Model creation is executed with NEURON and includes morphology instantiation and the definition of sections’ biophysics and synapses. RSME implements a visual stimulation pattern module, in which inputs such as expanding/collapsing rings and moving bars are provided to the network as structured light patterns. The model is simulated using NEURON’s solver, and the results are retrieved for analysis.
(EPS)

S2 Fig Synaptic input definition and biophysical specifications. **a.** An example synapse density pattern on a reconstructed SAC (Neuromorpho.org; ID: NMO_50993), where synapses are defined to 30% of the proximal sections (dark red shaded area), 10% of the distal section (light red shaded area), and linearly distributed in between. Synapse density is provided in an XML file, which is parsed and implemented by RSME. **b.** Example distributions of section’s passive conductance, reversal potential (g_pas_, e_pas_), and conductances of potassium, sodium and calcium channels (g_kdr_, g_kv3_, g_Nav1.8_, Cai). Values are according to SAC biophysical properties in [11]. Parameter distributions are specified in XML using SBML and parsed within RSME, which provides distributed mapping of the parameters and visualization.
(EPS)

S3 Fig Cell morphology and arrangement. (Top) Soma plots for a SAC population comprised of two grids (containing 16 and 9 SACs) (left) and a single DSGC (right). Somata are indicated with dots and colored boxes indicate the size of the cell, demonstrating the overlapping regions between cells and the two grids arrangement of the cells. (Bottom) full morphological plots for the SAC network (left) and DSGC (right).
(EPS)

S4 Fig SAC passive properties. **a.** Model of a 3D reconstructed SAC (Neuromorpho.org; ID: NMO_139062). **b.** (Left) Different current injections and the corresponding voltage deflections for a simulated SAC which morphology is shown in **a**. (Right) The change in voltage as a function of the current that was injected to the simulated SAC; from the slope of this curve we can extract the input resistance of the cell (84 MΩ). **c.** The capacitance (left), time constant (middle) and resistance (right) of recorded SACs (N = 24). Values were extracted from the voltage response of the cells to a pulse of -5 pA during recordings.
(EPS)

S5 Fig Genetic Algorithm parameter search. **a.** Initiation of the genetic algorithm population with 100 randomly selected individuals, each with eight parameters (see Methods). **b.** Illustration of the second generation. The best individuals from Gen 0 are copied to Gen 1 and the rest are a combination of different individuals from the previous generation (illustrated by individuals with combinations of colors). Random jitter (mutations) was added to some of the individuals. **c.** Same as in b for the X^th^ generation. **d-f.** Progression of GA maximization of the different objectives, as a function of generation number: the difference between the maximal voltage during the centrifugal and centripetal stimuli: Amp_CF_-Amp_CP_ (**d**); the difference between the rise time during the centripetal and centrifugal stimuli: RT_CP_-RT_CF_ (**e**); and the difference between the maximal voltage during the centrifugal stimulus and -10 mV. If the maximal voltage was below -10 mV, this objective was set to zero. This criterion was meant to prevent non-physiological depolarizations above -20 mV (**f**). Higher values indicate a stronger centrifugal preference. **g-i.** Same as (**d-f**) but with the reversed input kinetics. Here, no CF preferring SACs were found, as reflected by the rise time differences in **h** and the response amplitude difference in **g**.
(EPS)

S6 Fig A scatter matrix of parameters from the genetic algorithm and convergence analysis. The genetic algorithm was initiated to have a uniform distribution of parameters on all eight dimensions. In the lower left half of the figure, the first generation shows the extent of these in blue, and in green are the parameters in the 1^st^ to 45^th^ generation, which resemble SACs in their input-output characteristics (see Methods). The upper right half of the figure illustrates the extent of middle 80% of the cells from every 9 generations. Here we see that already after the first 9 generations, the region of solutions stabilizes, and doesn’t continue to significantly change over the generations of the genetic algorithm. This suggests that the genetic algorithm has found a region of the parameter space which is well-performing. The two example SACs from Fig 3 are also depicted. Note that their anatomical transition point and scaling factor parameters reside outside the high-density region of parameter space where most well performing cells are. This is due to the biological constrains which were not considered by the algorithm. As specified in the *Results*, all our example SACs met the biological criteria: higher density of excitatory inputs at ~70% of the proximal dendrites (anatomical transition point), and a significant difference between proximal and distal input densities (scaling factor).
(EPS)

S7 Fig Effect of SAC-SAC inhibition depends on the density of excitatory inputs in SAC distal processes. **a-d.** Two example SACs with similar excitatory input kinetic distribution and different input densities are affected differently by reciprocal inhibition between SACs. **a.** Left: input density of excitatory synapses. Right: Kinetics of synaptic input color coded by distance from soma. **b.** Voltage waveforms in response to CF (red) and CP (blue) motion, with increasing reciprocal inhibition. **c, d.** Same as **a** and **b** for a different SAC. Note that while the SAC in (**a, b**) reduced its CSI with inhibition weight, the SAC in (**c, d**) maintained positive CSI values. **e.** CSI values vs. density of excitatory inputs in the distal dendrites (given in synapse/μm). The simulation predicts that weak reciprocal inhibition in the SAC network enhances SAC CF preference in terms of response amplitude. When inhibition is strengthened, this enhancement is not increased, and a portion of the cells even reveal a decrease of CSI value. This decrease results from a suppression of the response amplitude due to the strong inhibition (**b**). Here, we show that SACs that have sparse inputs in the distal dendrites tended to maintain a positive CSI, while SACs that have more dense inputs in the distal dendrites tended to reduce their CSI. Note that according to EM studies the input density in the distal dendrites does not go down to zero [11,24].
(EPS)

S8 Fig Reciprocal inhibition is not sufficient to evoke centrifugal preference in non-CF preferring SACs. **a.** *Left*: Probability of excitatory synapses as a function of distance from soma for an example simulated SACs that did not display CF preference. *Center*: the color-coded kinetics of the simulated SAC excitatory inputs along the processes. *Right*: the voltage recorded from the soma of the simulated SAC in response to expanding (red) and collapsing (blue) rings. The CSI and RTI values are noted on the right. **b, c.** The CSI and RTI values of a population of non-CF preferring SACs (n = 12).
(EPS)

S9 Fig DSGC spiking properties. **a.** Example intracellular current-clamp recordings from a DSGC in response to presentation of a 2-sec bright spot (100 μm diameter). One repetition is shown in black, superimposed on nine other repetitions in grey. The Blue horizontal line denotes the baseline voltage of the cell (average of all traces), and the red line indicates the threshold for spiking. **b.** Average waveform of all cells (n = 15 DSGCs), aligned to the peak of the first spike evoked in response to spots presentation. **c, d.** Same as (a, b) but with gabazine application (5 μm; n = 10 DSGCs). Note that while resting potential increased in the presence of the GABA blocker, the spike threshold did not change.
(EPS)

S1 Data Source data SI_EphysData. Electrophysiology data recorded from SACs and DSGCs.
(ZIP)

S2 Data Source data SI_DSGC_simulation. Data from simulation of a DSGC embedded in a SAC network.
(ZIP)

S3 Data Source data SI_GA_SingleSAC&Network. Data from simulation of SACs generated by the genetic algorithm and of SAC responses within a SAC network.
(ZIP)
